# Between-the-Holes Cryogenic Cooling of the Tool in Hole-Making of Ti-6Al-4V and CFRP

**DOI:** 10.3390/ma14040795

**Published:** 2021-02-08

**Authors:** Asif Iqbal, Guolong Zhao, Juliana Zaini, Munish Kumar Gupta, Muhammad Jamil, Ning He, Malik Muhammad Nauman, Tadeusz Mikolajczyk, Danil Yurievich Pimenov

**Affiliations:** 1 Faculty of Integrated Technologies, Universiti Brunei Darussalam, Jalan Tungku Link, Gadong BE 1410, Brunei; asif.asifiqbal@gmail.com (A.I.); juliana.zaini@ubd.edu.bn (J.Z.); malik.nauman@ubd.edu.bn (M.M.N.); 2 College of Mechanical & Electrical Engineering, Nanjing University of Aeronautics & Astronautics, 29-Yu Dao Street, Nanjing 210016, China; zhaogl@nuaa.edu.cn (G.Z.); engr.jamil@nuaa.edu.cn (M.J.); drnhe@nuaa.edu.cn (N.H.); 3 Key Laboratory of High Efficiency and Clean Mechanical Manufacture, School of Mechanical Engineering, Shandong University, Jinan 210016, China; 4 Department of Automated Mechanical Engineering, South Ural State University, Lenin Prosp. 76, 454080 Chelyabinsk, Russia; danil_u@rambler.ru; 5 Department of Production Engineering, UTP University of Science and Technology, 85-796 Bydgoszcz, Poland; tami@utp.edu.pl

**Keywords:** cryogenic cooling, titanium alloy, drilling, composite material, liquid nitrogen

## Abstract

Lightweight materials are finding plentiful applications in various engineering sectors due to their high strength-to-weight ratios. Hole-making is an inevitable requirement for their structural applications, which is often marred by thermal damages of the drill causing unacceptable shortening of tool life. Efficient cooling of the tool is a prime requirement for enhancing the process viability. The current work presents a novel technique of cooling only the twist drill between drilling of holes with no effect of the applied cryogenic coolant transferred to the work material. The technique is applied in the drilling of two commonly used high-strength lightweight materials: carbon fibers reinforced polymer (CFRP) and an alloy of titanium (Ti-6Al-4V). The efficacy of the cooling approach is compared with those of conventionally applied continuous cryogenic cooling and no-cooling. The effectiveness is quantified in terms of tool wear, thrust force, hole quality, specific cutting energy, productivity, and consumption of the cryogenic fluid. The experimental work leads to a finding that between-the-holes cryogenic cooling possesses a rich potential in curbing tool wear, reducing thrust force and specific energy consumption, and improving hole quality in drilling of CFRP. Regarding the titanium alloy, it yields a much better surface finish and lesser consumption of specific cutting energy.

## 1. Introduction

Hole-making in alloys and composites is a primary requirement of their structural applications. Drilling, by far, is the most commonly used method for creating holes. Just like other machining processes, drilling also generates process heat, which should be efficiently dissipated to keep the progress of tool wear in check and obtain cutting of high-quality holes [1]. Unlike other processes, heat dissipation in drilling is highly cumbersome as the cutting area is totally enclosed when the drill is working inside an in-process hole and no direct access is available for a cutting fluid to absorb the heat [2]. The situation is even more troublesome in drilling of high-strength materials because they are cut with an expense of higher cutting energy and, thus, generate stronger heat flux in the cutting regions. Application of cryogenic cutting fluids have yielded positive outcomes, but the ratios of the fluid volume consumed to the amount of heat dissipated are considered too high to make the process viable. Understandably, the ratios are unacceptably high due to the lack of the fluids’ access to the cutting lips of the tool. The infelicitous situation calls for an innovative way of applying cutting fluids for the sake of more efficient heat dissipation and better drilling viability.

Ti-6Al-4V alloy and carbon fiber reinforced polymers (CFRP) are the most commonly used engineering materials possessing high strength-to-weight ratios. Their high resistance to corrosion makes them even more attractive for the structural applications. Both materials undergo cutting of holes at a huge scale, globally. The materials find extensive applications in automotive, aerospace, aircraft building, and marine engineering sectors. The high strengths and large-scale applicability of the materials make them ideal for carrying out investigations regarding effectiveness of cryogenic cutting fluids and their modes of application. A brief review of the published literature is provided below in respect of the application of cryogenic fluids in drilling of titanium alloys and CFRPs.

Cryogenic cooling involves application of a fluid operating at a temperature lower than −150 °C to the cutting zones in order to efficiently dissipate process heat. The most commonly utilized cryogenic fluid in the machining domain is liquid nitrogen (LN_2_) whose working temperature is around −196.5 °C. An experimental investigation was carried out to study the effects of applying LN_2_ in drilling of a titanium alloy using an inserted carbide twist drill. It was found that the application of the cryogenic fluid resulted in temperature drop effectively on one hand but degraded the hole quality on the other [3]. Another study has investigated the effects of using iced water flow as a heat sink near the drilling region to extract process heat [4]. The technique has caused improvements in working temperature, grain size, thrust force, and torque. The dissipation of process heat caused by the application of LN_2_ is reported to have significantly reduced tool wear leading to a positive gain in cutting speed [5]. The cryogenic fluid has also caused reduction in consumption of cutting energy in comparison with an emulsion-based coolant. Significantly larger reduction in cutting temperature leading to a better control of tool wear in comparison to that caused by no-fluid and flood coolant is also reported in another work [6]. The authors have also emphasized upon the milder environmental impact caused by the application of a cryogenic fluid. Application of CO_2_, as a cryogenic fluid, is also reported to have positive impacts on machining viability [7]. The tool life prolonged by the application of liquid CO_2_ is caused by reduction in chipping and crack propagation. An increase in flow rate of LN_2_ is found to have prolonged the tool life [8]. The authors have also reported improvement in surface integrity of the work material caused by increasing the pressure and flow rate of the fluid. Cryogenic cooling of coated carbide cutters using LN_2_ and compressed CO_2_ gas in side-and-end milling of Ti-6Al-4V is investigated [9]. The authors have concluded that cryogenic cooling applied to milling of the titanium alloy is not as effective as is to its turning because of rapid and periodic heating and super-cooling of the end mills’ teeth. Ahmed et al. have compared the performances of cryogenic cooling (LN_2_) and emulsion cooling in respect of cutting temperature, thrust force, dimensional accuracy, and surface roughness when applied to the drilling of ASTM B265 Grade 2 titanium alloy [3]. The authors found superiority of the cryogenic coolant in respect of all the performance measures except dimensional accuracy of the drilled holes. Hybridization of cryogenic cooling and minimum quantity of lubrication (MQL) is investigated for effectiveness in face-turning of a Ti-6Al-4V rod [10]. LN_2_-MQL and CO_2_-MQL hybrid lubro-coolants yielded highly favorable results regarding tool wear and surface finish, respectively.

Supply of chilled air in the drilling of CFRP is reported to have caused about 13%, 10%, and 7% reductions in the delamination factor, surface roughness, and acoustic emissions, respectively [11]. Another study has reported a reduction of about 19% in delamination factor with the application of chilled air in drilling of CFRP [12]. Dry drilling and drilling under LN_2_ cooling have been put up for comparison in hole-making of CFRP plates [13]. The cryogenic coolant is reported to have caused significant improvements in surface roughness and delamination whereas the holes’ dimensional accuracy had marginally improved. A cryogenically treated CFRP specimen is tested for drilling performance [14]. It is found that although the technique has yielded better surface finish but has also caused an escalation in thrust force and intensification in delamination. Another study has reported that an extremely low temperature caused by LN_2_ causes shortening of tool life and increase in thrust force [15]. Raj et al. have tested strengthening of a twist drill material by treating it at a low temperature using a cryogenic fluid [16]. The authors have reported reductions in intensity of tool damage, surface roughness, and exit hole delamination. The effects of cryogenic cooling are compared with those of dry cutting in drilling of thermosets and thermoplastics based CFRP composites [17]. The cryogenic coolant caused reductions in fiber pull-out and delamination at the exit side of holes in respect of thermosets based CFRP and cut down the variations in hole dimensions regarding the thermoplastics-based composite. An experimental investigation carried out on rotary ultrasonic machining of CFRPs being performed in a cryogenic environment concludes that an increase in cutting speed results in a reduction in thrust force because of an abatement in the axial stress [18]. A study carried out on quantifying the effects of drilling parameters and cryogenic cooling on the performance measures of hole-making in glass fiber reinforce epoxy composites suggests that the cryogenic cooling significantly increases microstructural hardness and reduces delamination factor [19]. It is further reported that the cutting speed also needs to be fine-tuned along with the supply of a cryogenic fluid for achieving substantial reductions in surface roughness of the drilled holes. In drilling of GLARE (glass laminate aluminum reinforced epoxy) laminates, the application of LN_2_ is reported to have reduced the formation of exit burr by about 47% in comparison with dry drilling [20].

The review provided above suggests that cryogenic fluids are either supplied in a continuous manner or just once before the start of the drilling process. Direct inaccessibility of the fluid to the cutting lips while the drill is operating inside a hole gives rise to yet another possibility of fluid’s supply. The current work investigates the efficacy of supplying a cryogenic fluid only to the drill, without getting in contact with the work material, before cutting each hole of a run. The motivation behind the idea is to avoid hardening of the work material while cooling and strengthening the drill’s body using the coolant. The novel approach of cryogenic cooling is also compared with continuous cryogenic cooling and no-cooling in drilling of CFRP and Ti-6l-4V plates. The performance measures quantified in this regard are tool wear, thrust force, surface roughness, specific cutting energy, productivity, and consumption of the cryogenic fluid.

## 2. Materials and Methods

This section provides details on the work materials, parameters (controlled, measured, and fixed), design of experiments, experimental setup, tooling, and measurements.

### 2.1. Work Materials and Tooling

The performance of the novel cryogenic cooling approach is tested upon drilling of a high-strength alloy and a composite material. Their pertinent details are provided below. The composite material is T700G, a bi-directional 0°/90° weaved carbon-fiber composite plate. Thirty three plies are stacked up to yield 22.5 mm thickness of the CFRP plate. T700S high-strength non-twisted carbon yarns of 7 µm diameter are packed into Rhino 1411 epoxy resin. The density, tensile strength, and tensile modulus of the carbon fibers are 1.8 g/cm^3^, 4.9 GPa, and 240 GPa, respectively. The densities of the epoxy resin and the resulting composite are 1.104 and 1.38 g/cm^3^, respectively. The resin is so normalized to yield 60% of fiber volume.

A commonly used α + β alloy of titanium, Ti-6Al-4V is the alloy tested in this work. The dimensions of the work material plate are 100 mm × 200 mm × 19 mm. The annealed form of the work material is obtained by holding the soaking temperature of the plate between 777 and 783 °C for about 70 min followed by air cooling. Table 1 presents the mechanical properties of the work materials.

The drilling tools used in the 12 runs are FIREX (multiple layers of TiAlN and TiCN) coated tungsten carbide stub drills. The ceramic coating imparts the features of extra heat resistance and shock resistance to the drills. The drills possess the number of flutes, point angle, cutting diameter, total length, and fluted length of 2, 140°, 8 mm, 79 mm, and 41 mm, respectively. Each drill is held in a collet 45 mm away from the chisel edge before being clamped in the tool holder.

### 2.2. The Parameters

The following two parameters are controlled for each of the two work materials:

1. Cooling option. The following three techniques are tested for each of the two work materials: (a) Dry drilling (no cutting fluid is used); (b) Continuous cooling using a jet of LN_2_; and (c) Between-the-holes cooling using LN_2_.
2. Cutting speed (*V*_c_). The low and high levels of this parameter are 100 and 150 m/min and 13 and 20 m/min, respectively, for CFRP and Ti-6Al-4V.

The aforementioned control parameters and their levels result in six (=3 × 2) experimental runs for each of the two work materials. The experiments are run in a single replicate. The two levels of cutting speed for each of the two work materials are finalized after performing preliminary runs. A cutting speed in excess of 20 m/min resulted in complete rupture of drills in hole-making of the titanium alloy. For CFRP, a cutting speed beyond 150 m/min caused a jump in tool’s abrasion and surface deterioration around the drilled holes. Therefore, the high levels of the predictor are fixed to these values of cutting speed so as to ensure viable production rates. Each of the 12 experimental runs involves drilling of five 8 mm diameter thru-holes using the same twist drill. A new twist drill is used for each run. The following parameters (response variables) are determined for each experimental run.

1. Tool damage, measured as the average width of flank wear land developed on the lips of the twist drill, *VB* (µm), to be evaluated after drilling five holes in the work material;
2. Thrust force, *F*_z_ (N), averaged for the five holes;
3. Average surface roughness of the holes, *R*_a_ (µm);
4. Specific cutting energy, *SCE* (J/mm^3^), averaged for the drilling of five holes;
5. Production time for cutting five holes, *t*_p_ (sec);
6. Volume of the cryogenic fluid consumed in cutting five holes, *FC* (L).

The feed rate for all the runs is fixed to 0.1 mm/rev leading to various feed speeds for the different levels of cutting speed. The CNC machining center’s rapid traverse (15,000 mm/min) is utilized to retract the drill when it completes the cutting stroke, lift it to the tool-only cooling position (for the runs involving between-the-holes cooling), and move to the next hole’s location. Equations (1) and (2) present the formulae for calculating the production times:

(1) $$t_{p}(Dry/Continuous)=\left[\left(\frac{h}{V_{f}}\right)+t_{RP}\right]\times 5$$

(2) $$t_{p}\left(Between-the-holes\right)=t_{p}(Dry/Continuous)+5t_{s}$$

where *h*, *V_f_*, 5, *t_RP_*, and *t_s_* are depth of hole, drill’s feed speed, number of holes drilled per run, time for retracting and positioning the tool, and time consumed in jetting the cryogenic fluid on the tool before initiating cutting of each of the five holes, respectively. *t_RP_* is fixed to 0.6 s, whereas the values of *t_s_* are equal to 4 and 8 s for the hole-making of CFRP and Ti-6Al-4V, respectively.

### 2.3. Experimental Setup and Measurements

**Figure 1:**
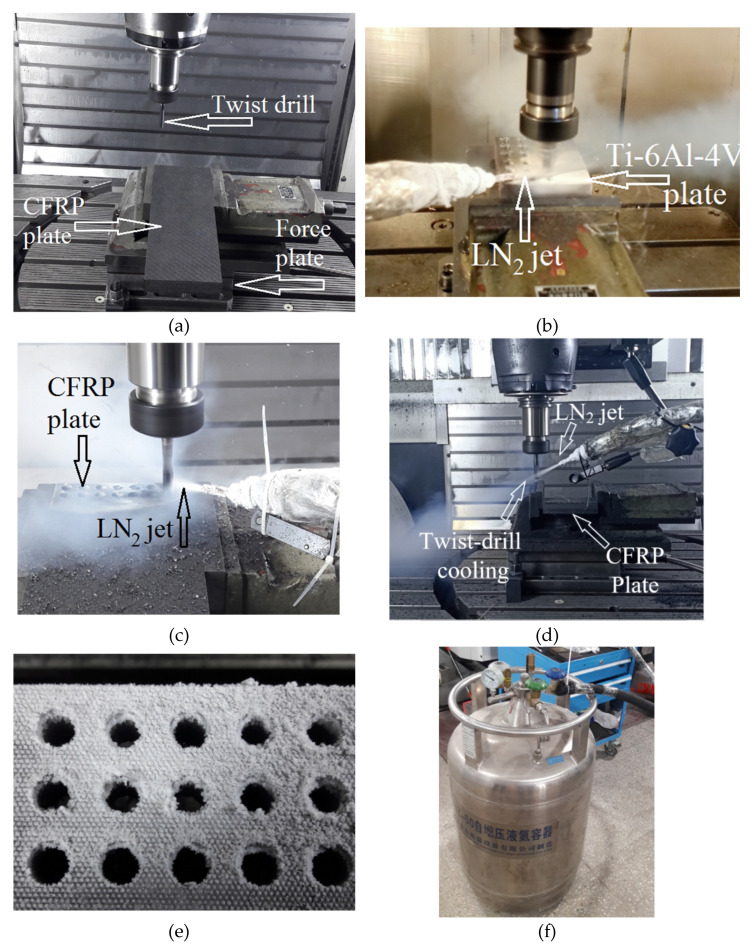
Experimental setup: (**a**) dry drilling of CFRP; (**b**) continuous cryogenic cooling in drilling of Ti-6Al-4V; (**c**) continuous cryogenic cooling in CFRP drilling; (**d**) between-the-holes cryogenic cooling in CFRP drilling; (**e**) CFRP plate just after finishing a run employing continuous cryogenic cooling; and (**f**) LN_2_ dewar.

All the experiments are performed on a 5-axis CNC machining center, DMG Mori DMU 60 having maximum motor power and spindle speed of 25 kW and 20,000 rpm, respectively. All the runs involving CFRP are performed before those of Ti-6Al-4V. Although the thicknesses of the CFRP and Ti-6Al-4V plates are 22.5 mm and 19 mm, respectively, the tool is fed to the depths of 24.5 mm and 21 mm, respectively, to ensure complete exit of the cutting lips at the exit sides of the plates. For the runs involving continuous and between-the-holes cryogenic cooling, the LN_2_ is supplied from a storage dewar at a flow rate of 0.5 L/min through a thermally insulated pipe fitted with a nozzle of 6 mm diameter on its delivery end. For continuous cooling, the nozzle is so directed to have the maximum impingement of the fluid’s jet on that part of the drill’s body which is rotating just outside the surface of the in-process hole. The between-the-holes cooling technique is realized by rapidly lifting the rotating drill up to a height of 15 cm from the work surface and impinging it with the jet of LN_2_ for exactly 4 and 8 s related to the drilling of CFRP and Ti-6Al-4V, respectively. The lifting of the drill prior to the jet impingement is carried out to ensure complete avoidance of work cooling through the cryogenic coolant. The cooling process is followed immediately by a rapid displacement of the drill to a location close to the point of drilling next hole on the work surface. The regular feed speed of the drill commences thereon for cutting the hole. Figure 1 shows the experimental setup.

The tool damage is measured using a camera-fitted optical microscope, ARTCAM 130-MT-WOM. The used drills are cut at a distance of about 15 mm from the chisel edges using wire electric discharge machining and are cleaned in an ultrasonic cleaner prior to the measurements. The *VB* is determined by averaging the width of the flank wear land measured at four different locations of each of the two lips. A portable roughness tester, Mahr Perthometer M1, is used to measure the average arithmetic roughness (*R*_a_) of the holes. The stylus of the instrument scans the surface for a distance of 5.5 mm and the procedure is applied at three different locations of each of the five holes drilled in an experimental run. The *R*_a_ is then determined by averaging the 15 readings. The thrust force is measured using Kistler piezoelectric dynamometer 9265B utilizing a force plate 9443B. The range of force measurement for the instrument along the direction of drill’s feed (*z*-axis) is 0–30 kN. The force–time data obtained from the force measurement system is processed in Origin (OriginPro 2015 SR2), a computer program for interactive scientific graphing and data analysis. The processing returns a numbered value of the force data as *F*_z_, which is most central to the force-time graph obtained for the length of actual cutting.

Specific cutting energy is determined through the measurements of electrical current drawn in by the CNC machine tool. Three current clamp meters, Hantek CC 65, are applied to the three phases of the CNC machine’s power bus. The total power drawn in by the machine tool is determined by using the formula:

(3) $$P_{\mathrm{total}}\;=\;\frac{\sqrt{3}.PF.V\left(I_{1}+I_{2}+I_{3}\right)}{3}$$

where *V* and *PF* represent potential difference (in volts) and power factor, respectively. *I*_1_, *I*_2_, and *I*_3_, quantified in Amperes, are the current readings of the three clamp meters. Non-cutting powers for the four levels of cutting speed (13, 20, 100, and 150 m/min) are measured by rotating and vertically moving down the twist drill in the air at the given combinations of cutting speed and feed speed. The drill is moved at the feed speeds of 51.7, 79.6, 398, and 597 mm/min against the cutting speeds of 13, 20, 100, and 150 m/min, respectively, ensuring the fixed feed rate of 0.1 mm/rev. The four feed speeds yield the material removal rates (*MRR*) of 43.3, 66.8, 333.4, and 500 mm^3^/sec, respectively. The average cutting power for each run is found by subtracting the respective non-cutting power from the corresponding total electric power consumed by the equipment. The average cutting power for each run is then divided by the corresponding *MRR* to get the *SCE*.

## 3. Results

This section presents the experimental results, analyses, and discussions regarding the response variables listed in the previous section.

### 3.1. Tool Damage

**Figure 2:**
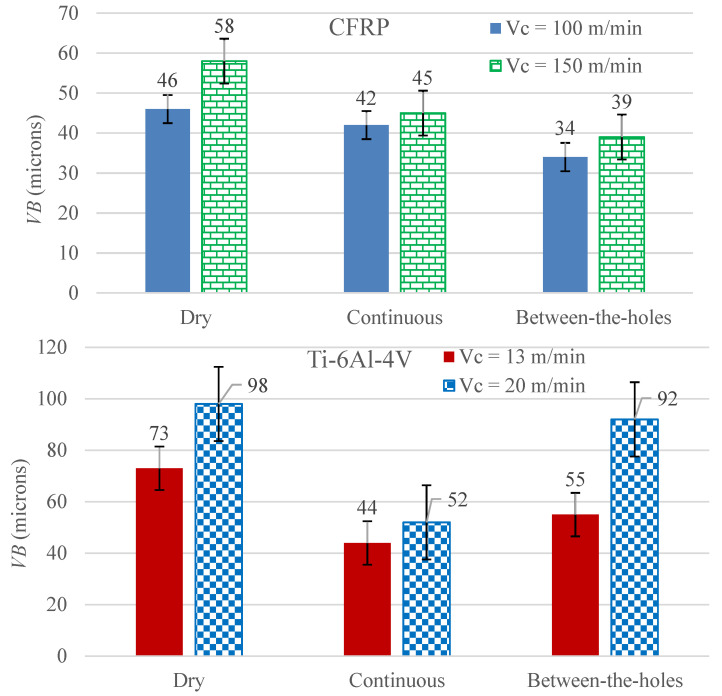
The measurements of the drills’ *VB* after cutting 5 consecutive holes.

Figure 2 presents the experimental results regarding *VB*, categorized by the two work materials tested (CFRP and Ti-6Al-4V). Two inferences, common to both the materials, can be directly drawn from the data bars. Firstly, the high level of cutting speed causes rapid deterioration of the cutting lips. Secondly, the application of the cryogenic coolant (LN_2_) does inhibit the progress of tool wear.

It is clear from the data presented in Figure 2 that the novel between-the-holes cooling technique is better than the traditional continuous cooling approach for the case of CFRP only, although it still outperforms dry drilling in the cutting of Ti-6Al-4V. The difference in respect of the mode of the coolant’s application is so significant for both the work materials that the *VB* of the favorable mode related to the high level of cutting speed is lower than the *VB* of the non-favorable mode associated with the low level of cutting speed.

Application of LN_2_, due to its extremely low working temperature, puts a curb on the progress of tool wear by quashing the heat flux and maintaining the hardness of the twist drill’s cutting lips. Even at the full depth of the drill inside the hole, as is the case with the continuous mode of cooling, a noticeably high thermal conductivity of tungsten carbide (110 W·m^−1^·K^−1^) causes efficient heat dissipation from the cutting area because of a steep temperature gradient existing between the high temperature cutting lips and the considerably colder drill’s body rotating outside the hole and being impacted directly with the LN_2_. The sustained hardness of the cutting edges and the adjacent faces keeps up the wear resistance against the abrasion of the hard carbon fibers of CFRP or hard phases of Ti-6Al-4V matrix. As is visible in images (b), (c), and (e) of Figure 1, the continuous cooling mode results also in impingement of the work surface with the cryogenic fluid’s jet. The resulting exposure of the work material to a supercool fluid tends to raise its strength. The strengthening tendency has a different outcome in respect of the actual increase in hardness based on the composition, macro-/micro-structure, and thermal characteristics of the work material. As Ti-6Al-4V is a metallic alloy, its thermal conductivity is much higher than that of a ceramic reinforced polymer matrix composite, such as CFRP (7.2 compared to 0.9 W·m^−1^·K^−1^). The higher conductivity of the titanium alloy quickly disperses any cooling/heating effect throughout the material’s body, thus, diminishing any localized hardening/softening effect. Hence, it can be safely said that the continuous cooling technique does more good than bad to the drilling process by effectively removing the process heat and maintaining the tool’s hardness during cutting while not causing any significant work hardening. The between-the-holes cooling technique in drilling of the titanium alloy does not provide much needed cooling during the cutting process. The pre-cutting cooling of the drill is not enough to take on the high rate of heat generation and associated softening and wearing of the tool during cutting of the high-strength titanium alloy. The discussion, thus, establishes that the between-the-holes mode of cryogenic cooling is not as effective as the continuous mode in drilling of Ti-6Al-4V.

As CFRP is not a good thermal conductor, the LN_2_ jet impacting the work material close to the cutting area, in the continuous cooling mode, significantly cools it down because the cooling effect cannot be dispersed efficiently through the body. The resulting cooling effect raises the strength of the composite, especially of the carbon fibers, around the hole being drilled. Consequently, the hole is cut with a higher severity of abrasion leading to an increase in the *VB*. On the other hand, the between-the-holes mode does not offer any cooling to the work material that might otherwise cause it to gain strength. The cryogenically cooled drill, prior to the start of hole cutting, remains hard enough to cut the hole in a composite material whose major portion consists of a soft polymer matrix. Furthermore, as the process of hole-cutting carried out on CFRP is exceedingly faster than Ti-6Al-4V (see the relevant feed speeds in Section 2.3), the drill does not lose much of its coldness to the surroundings. On these grounds, it can be stated that the between-the-holes mode of cryogenic cooling is more effective in curbing *VB* than the continuous one in drilling of CFRP.

The effect of cutting speed is as expected. Its high level causes rapid deterioration of cutting lips due to progressive abrasive wear and other temperature dependent modes of tool damage. An increase in cutting speed causes an increase in the rate of process heat generation leading to intensification of heat flux around the cutting lips. The accumulation of the process heat causes softening of the cutting edges—leading to a higher wear rate—and escalation of work adhesion on the adjacent faces (rake and flank). Both forms of the tool damage seriously cut short the useful life of the twist drill.

**Figure 3:**
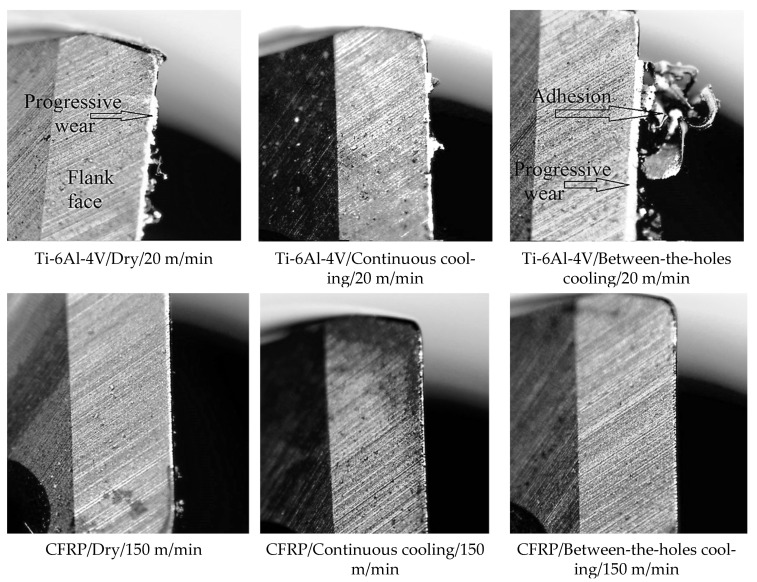
Optical micrographs of the cutting edges and flank faces of the drills used in the selected runs

Figure 3 presents the optical micrographs of the cutting lips of the drills used in the selected runs. These images are taken prior to the cutting and ultrasonic cleaning of the drill bits. To have fair mutual comparisons, only the drills used in the runs employing the high level of cutting speed (20 m/min for Ti-6Al-4V and 150 m/min for CFRP) are presented in the figure. There is a marked difference between the damage modes sustained by the drills in cutting Ti-6Al-4V and CFRP. The cutting of the former shows the impacts of progressive wear as well as strong adhesion whereas the latter only shows the signs of progressive wear (bright vertical lines on the cutting edges).

Ti-6Al-4V is an alloy that possesses a high degree of adhesivity, especially at elevated temperatures, with most of the tool materials. Adhered particles of work material damage the tool’s geometry by taking minute flakes of the tool material with themselves as they chip off from the tool’s surface under the machining stresses. Considerably higher degrees of adhesion are visible in the first and the third images of Figure 3 as compared to the second, which suggest that drilling of the titanium alloy under a continuous supply of LN_2_ keeps a check on adhesion due to the effective lowering of working temperature around the cutting lips. Pre-drill cooling with a curtailed supply of the cryogenic coolant during cutting does not help in abating adhesion. Furthermore, it is also visible that the levels of abrasive wear endured by the drills in cutting the titanium alloy are higher than in cutting the composite material. Although, carbon fibers are much harder than the titanium-based phases, the presence of a soft epoxy matrix soothe things down for CFRP drilling. The three micrographs related to the drilling of CFRP show mild progressive wear and no signs of adhesion. Furthermore, the micrograph related to the dry cutting shows a wider flank wear land than the ones employing cryogenic cooling. The observation suggests that the application of the cryogenic fluid does help the cutting lips to maintain their wear resistance by effectively removing the process heat.

### 3.2. Thrust Force

**Figure 4:**
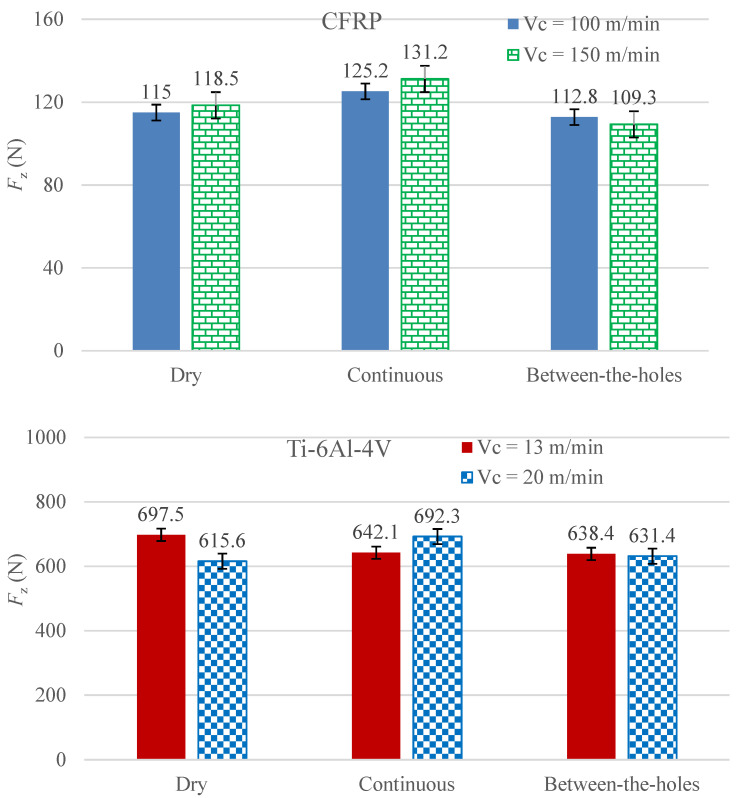
The measurements of thrust force averaged for the cutting of five holes.

As drilling is a uniaxial cutting process, the two planar and mutually orthogonal components of the cutting force, *F*_x_ and *F*_y_, are not taken as the process’s performance measures. The two are exceedingly smaller in magnitude than the thrust (axial) component, *F*_z_, and are, thus, not analyzed. Figure 4 presents the measurements regarding thrust force for the 12 experimental runs (six each for the two work materials). Clearly, between-the-holes cryogenic cooling is the best option for both the work materials. It outperforms the continuous mode of cryogenic cooling by avoiding work strengthening as the work material is not exposed to the coolant’s jet. Understandably, a stronger form of work material cuts with a higher level of thrust force. The between-the-holes mode of cooling yields smaller thrust forces than dry drilling because of the milder levels of tool wear associated with the runs employing the cooling technique. Milder tool wear means lighter blunting of the cutting edge, thus, requiring lesser cutting force to yield the same magnitude of stress needed to plastically deform the work material.

The thrust forces experienced in the drilling of the titanium alloy are exceedingly higher than in the composite material. The magnitude of cutting forces depends primarily on the shear strength of a work material; the higher the shear strength the higher is the magnitude of the cutting force required overcoming the strength and forming the chip. Table 1 shows that the shear strength of the titanium alloy is about five times as that of the composite material, explicating as to why the titanium alloy cuts with a higher thrust force despite having a lower tensile strength.

As can be observed from Figure 4, cutting speed brings about mixed effects on thrust force. Valid for both the work materials, an increase in cutting speed causes an increase in thrust force when the cryogenic coolant is applied during the cutting process, but an opposite effect is recorded when it is administered before start of the cutting process. A plausible explanation to this phenomenon is that an increase in cutting speed accelerates process heat generation intensifying the heat flux in the cutting region. The intensified heat flux raises the temperature of the work material adjacent to the hole being cut, causing a reduction in its flow stress (instantaneous yield strength). Consequently, a material with lower yield strength is cut with a lower magnitude of force. This phenomenon commonly known as thermal softening. On the other hand, if the work material is impinged upon with a supercool fluid during the cutting process, as is the case in the continuous cooling approach, the heat flux does not build up to an extent required to instigate thermal softening. Thus, an increase in cutting speed for the runs employing the between-the-holes cooling technique results in the lessening of thrust force.

**Figure 5:**
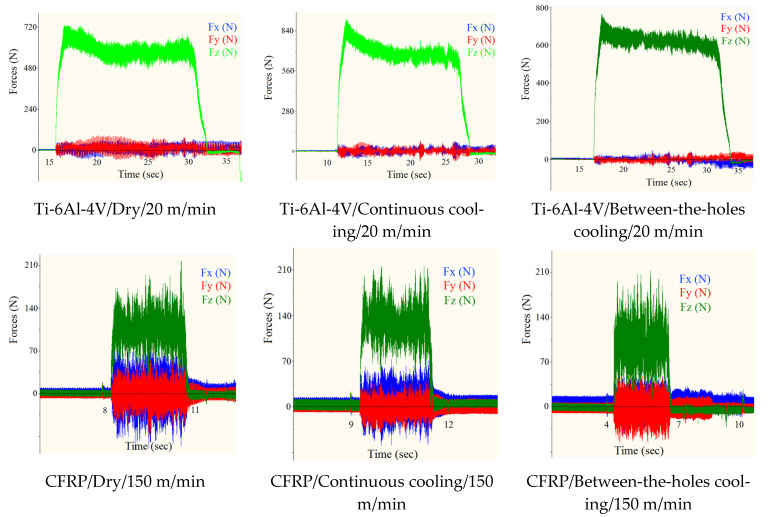
Variations in the machining force components with time in cutting hole number 3 for the selected runs.

Figure 5 shows variations in the machining force components with time for the six runs employing the high level of cutting speed in drilling the third of the five holes. The time lapses shown on the graphs related to CFRP are much shorter than those of the titanium alloy because of the difference between the cutting speeds, and consequentially between the feed speeds, employed for drilling of the two materials. The difference between the magnitudes of the planar components (F_x_ and F_y_) and the thrust component (F_z_) is exceedingly larger in drilling of Ti-6Al-4V. This is why thrust force is solely taken for analyses as it is the component which gets most affected by a change in the work material’s shear strength. The F_z_ curves related to Ti-6Al-4V show a downward trend as the cutting progresses with more prominence in the one related to the continuous mode of cryogenic cooling. The trend depicts thermal softening of the material occurring due to the intensification of heat flux as the drilling process progresses. The trend is more prominent in the graph related to continuous cooling because the work material is already strengthened a bit due to the impingement of the cryogenic fluid at the start of the cutting. Thereafter, the cutting heat steeply brings the thrust force down as the material quickly starts to soften down.

### 3.3. Surface Roughness of the Holes

**Figure 6:**
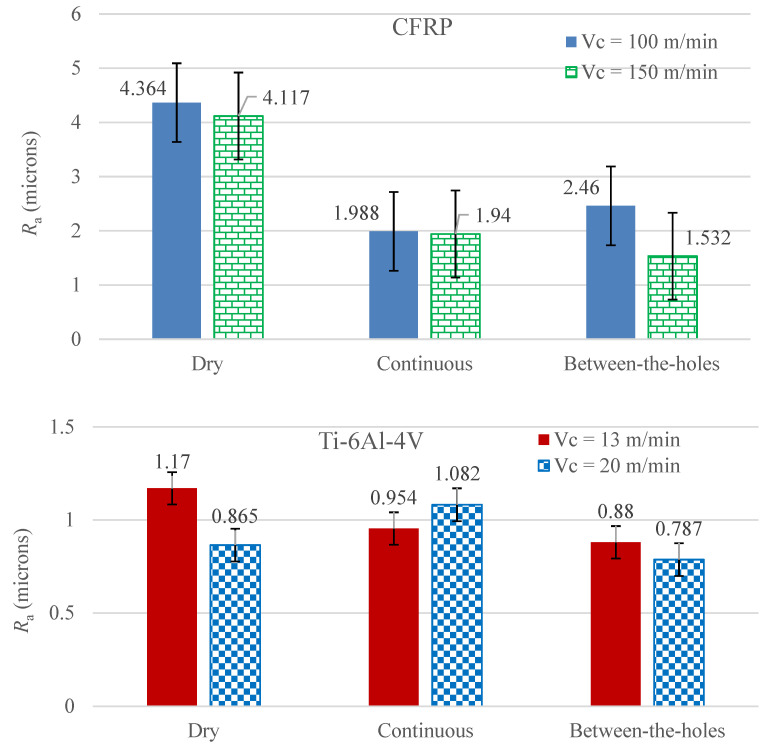
Results of average arithmetic surface roughness for the 12 experimental runs

Figure 6 presents the *R*_a_ measurements of the drilled holes for the 12 experimental runs. A few inferences can be easily drawn from the data bars. Firstly, the between-the-holes cooling approach is easily the best option for both the work materials as it collectively yields the lowest values of *R*_a_. Secondly, for CFRP, dry drilling yields far worse *R*_a_ results than the cryogenic cooling based options whereas its results are comparable with those of the continuous cooling mode for Ti-6Al-4V drilling. Thirdly, the high level of cutting speed yields lower values of *R*_a_ for all the material-cooling combinations except Ti-6Al-4V–continuous cooling combination. Fourthly, the surface finish of holes produced in the titanium alloy is exceedingly better than in the composite material. As CFRP is a heterogeneous material comprising very hard carbon fibers blended in a very soft epoxy matrix, the machining process is, generally, expected to yield poor surface finish.

Dry drilling yields poor surface finish because of the blunt cutting edges of the associated drills. Dry cutting causes intense tool wear, leading to loss of cutting edges’ sharpness, which in turn leads to the high values of *R*_a_. The supply of LN_2_ in a continuous mode does reduce the magnitude of tool wear causing improvements in surface roughness of the drilled holes. Unfortunately, the application of a cryogenic fluid in a continuous manner has a side effect as well. In addition to cooling the tool, the fluid also cools the work material. The cooling effect induced on the work material varies in magnitude depending upon the distance from the cutting area, cryogenic fluid’s impingement location, volume flow rate of the fluid, and thermal characteristics of the material. The simultaneous heating (because of cutting) and uneven cooling effects produce complex expansion and contraction patterns in the work material with respect to time, resulting in deterioration of the holes’ surface. The between-the-holes cooling approach is free of such a side effect. It not only keeps a check on the wear of the cutting edges, as described in Section 3.1, but also avoids the complex heating and cooling mechanism of the work material, leading to attainment of a better surface quality.

The high level of cutting speed is found to yield lower surface roughness. The improvement in surface quality is attributed to a faster cutting process which does not grant a time period long enough to allow a high proportion of process heat to be absorbed by the work body. Rather, a large proportion of the process heat is taken away by the chip. Consequently, a more mechanical and less thermal effect dispensed to the work surface results in the reduced values of surface roughness.

**Figure 7:**
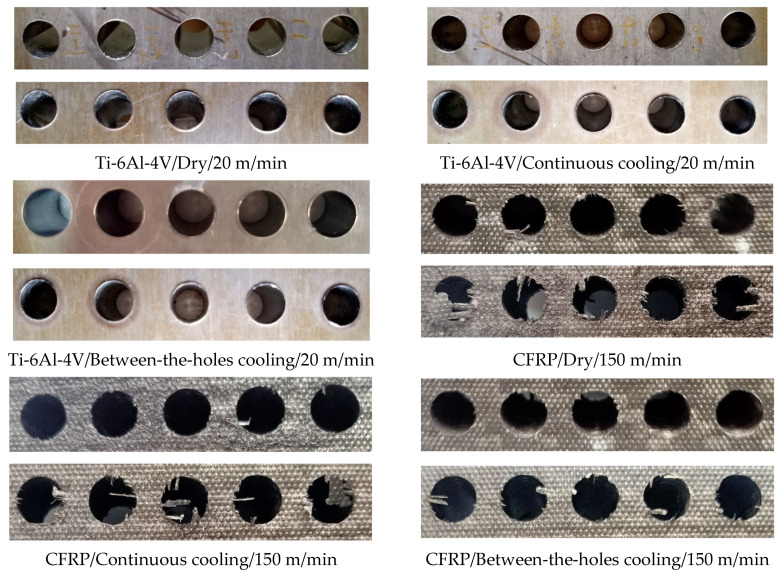
Images of the 5 holes drilled in each of the selected 6 runs. For the image of each run, the top and bottom rows show the entry and exit sides, respectively, whereas the sequence of holes cutting for both the rows run from left to right.

Figure 7 presents the images of the entry and exit sides of the holes drilled in the runs employing the high level of cutting speed. The sequence of cutting and the distribution of the entry and exit sides of the holes are described in the figure’s caption. Seemingly, the quality (roundness, absence of burrs, etc.) of the Ti-6Al-4V holes is much better than the CFRP ones. This is so because the titanium alloy is a homogeneous and hard stuff whereas CFRP is a heterogeneous combination of hard fibers held in a soft matrix. The behaviors of the two constituent materials in respect of thermal expansion, elastic behavior, and plastic deformation are entirely different, causing them to respond very differently to a common stimulus. As expected, the entry sides of the holes, for all the runs, are better shaped than the exit sides. Formation of burr harms the quality of holes on the exit side and is a common phenomenon observed in drilling of all the materials. As can be seen from the first 3 images of Figure 7, the effects of cooling option are not obvious on the geometric quality of the Ti-6Al-4V holes. The same is not the case with the CFRP holes. The quality of holes produced under the between-the-holes mode of cryogenic cooling is much better than the continuous mode and dry drilling. As described above, the between-the-holes cooling technique keeps the drill cool, hard, and less prone to wear on one hand and avoids complex expansion/contraction pattern of the work material on the other by not impacting it with the cryogenic fluid. The technique results in production of round holes in CFRP with milder intensity of burring and fiber pullout.

### 3.4. Specific Cutting Energy

**Figure 8:**
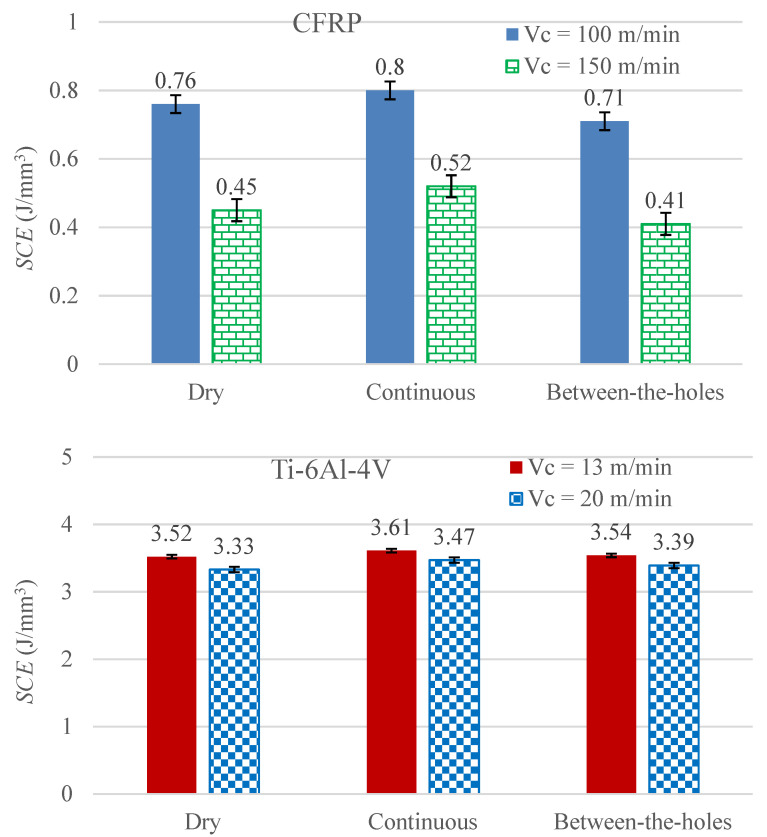
*SCE* measurements for the 12 experiments.

Figure 8 presents the results regarding *SCE* for the 12 experimental runs. *SCE* is the energy required at the cutting edges to remove a unit volume of work material. Its magnitude mainly depends on the shear strength of the work material. As the titanium alloy is much stronger in shear than the CFRP composite, the cutting of the former requires much larger consumption of specific energy than the latter. For both the work materials, an increase in cutting speed causes a reduction in consumption of specific cutting energy. The decreasing effect on *SCE* caused by a rise in cutting speed is attributed to the work material’s thermal softening, which is instigated by the resulting intensification of heat flux [21]. By increasing the cutting speed, the work material is machined with a disproportionally smaller increase in cutting power with respect to the corresponding increase in material removal rate (*MRR*) [22]. As *SCE* is formulized as the total cutting power divided by the *MRR*, an increase in cutting speed causes a reduction in the *SCE*.

The data bars related to both the work materials reveal that the *SCE* is increased when the cryogenic coolant is applied in the continuous mode. On the other hand, the between-the-hole mode of cooling causes a reduction in specific cutting energy consumption in the drilling of CFRP whereas a marginal increase is recorded for Ti-6Al-4V. The obvious reason associated with the unfavorable outcome yielded by the continuous mode is the simultaneous cooling and, thus, hardening, of the work material in addition to the twist drill. The supply of the coolant during the cutting process raises the strength of the material making it consume more energy for its plastic deformation and conversion into a chip [23]. The favorable outcome associated with the between-the-holes cooling when applied to the drilling of the composite material is the retention of the cutting edges’ sharpness, which does not let the *SCE* magnitude rise significantly. In a contrast, the same cooling technique has not yielded any *SCE* reduction in the drilling of Ti-6Al-4V when compared with dry drilling. The attributed reason is that although the work material’s mechanical behavior is same for the two cooling options (dry and between-the-holes cryogenic cooling), the drills’ deteriorated cutting lips in the case of between-the-holes cooling, as is obvious in Figure 2 (bars related to Ti-6Al-4V), have caused a growth in the *SCE* requirement.

### 3.5. Production Time and Consumption of Cryogenic Fluid

As described before, production time and volume of the cryogenic fluid consumed can directly be calculated from the settings of the input parameters. Their calculations for the 12 experimental runs are presented in Table 2. Understandably, dry drilling stands out with respect to the two measures as it does not use any cooling fluid, nor does it need to interrupt the process for intermittent cooling.

With respect to production time, continuous mode of cryogenic cooling is as good as dry drilling because it does not need to halt the tool movement to ensure the requirement of tool-only cooling. The performance of the between-the-hole mode of cooling depends upon the cutting speed used for the particular run and the time duration for which the jet is applied to the drill before cutting each hole. The cooling technique takes 40%, 61%, 127%, and 179% more time than dry drilling (and continuous cooling) to process 5 holes against the cutting speeds of 13, 20, 100, and 150 m/min, respectively. This implies that the higher the cutting speed the larger is the percentage difference between the between-the-holes cooling technique and the other two options regarding production time. As the high levels of cutting speed are associated with the drilling of CFRP, the between-the-holes technique may badly affect the productivity of hole-making in the composite material.

With respect to consumption of the cryogenic coolant (LN_2_), both of the non-dry drilling options incur an extra cost. Of the two, the better cryogenic cooling option is decided by the cutting speed and the time duration of the fluid’s application selected for the between-the-holes cooling mode. As described in Section 2.3, the time durations for the fluid’s application are 4 and 8 s, respectively, for the drilling of CFRP and Ti-6Al-4V carried out under the between-the-holes cooling approach. The fluid’s impingement period for the titanium alloy is kept double than that of the composite material because the drilling of the former is characterized by more intense heat flux and lower cutting speeds (and feed speeds). Under these settings, the continuous cooling technique consumes marginally more cryogenic fluid than the between-the-holes technique in the drilling of CFRP, but this difference swells to very high values in the cutting of the titanium alloy, as is observable from Table 2. The former consumes twice and thrice as much LN_2_ than the latter in the drilling of Ti-6Al-4V at the cutting speeds of 20 and 13 m/min, respectively. The analysis suggests that the between-the-holes mode of cryogenic cooling is an economical option as compared to the continuous mode when low-to-medium cutting speeds are involved, such as for the drilling of Ti-6Al-4V. Whether or not the between-the-holes technique of cooling is more economical than dry drilling needs a deeper cost analysis involving the acquisition costs of the twist drills and liquid nitrogen, electricity tariff, and tool life criterion.

## 4. Discussion

In order to establish the viability of the novel between-the-holes cryogenic cooling approach, the experimental results provided in Section 3.1, Section 3.2, Section 3.3, Section 3.4, Section 3.5 need to be discussed in a holistic manner. The idea exploited in realizing the cooling technique is to cryogenically cool the drill before the cutting of a hole to maintain its wear resistance while completely avoiding the impact of the coolant on the work so as to prevent its strengthening and contraction. The novel cooling technique is pitched against the conventional continuous mode of cryogenic cooling and no-cooling approach in drilling of a titanium alloy and CFRP composite. The performance of the cooling technique is comparatively analyzed with respect to its effects on tool damage, thrust force and cutting energy, hole quality, and productivity and volume of the cryogenic fluid consumed.

Dry drilling yielded comparatively favorable results regarding none of the performance measures analyzed in this work. The only supportive aspect of the no-fluid cutting is that it does not incur any cost related to consumption of a cryogenic coolant. It can, thus, be positively asserted that application of a cryogenic fluid, such as liquid nitrogen, possesses highly favorable outcomes with respect to drilling of the titanium alloy and CFRP. It then comes to finding the most effective mode of applying the cryogenic fluid that would return the best results in terms of the performance measures. Liquid nitrogen is conventionally applied in a machining process as an unceasing jet continuously targeting the cutting edges of the tool. Drilling is quite different from the other machining processes as the cutting edges of the tool remain accessible to the coolant for a very small portion of the cutting process. In such a situation, the continuous supply of a coolant is actually impinging the work material more than the cutting tool’s edges. It asserts that the conventional mode of cryogenic cooling affects the work material more than the drill. As most of the work materials respond to the impact of a cryogenic fluid by increasing their strengths, the outcome does not sit well with the objectives of cryogenic cooling. An increased work strength makes cutting difficult and leads to more intense tool wear, higher cutting forces, and consumption of more cutting energy. In this context, the technique of cryogenically cooling only the drill for some time and curtailing the supply of the coolant during cutting is put forward.

The experimental results on tool damage bring about different conclusions regarding the two work materials, Ti-6Al-4V and CFRP. The between-the-hole mode of the coolant supply causes better suppression of tool wear than the continuous mode in the drilling of CFRP. The opposite is true for the drilling of Ti-6Al-4V. As the titanium alloy is a better conductor of heat than CFRP, the cooling effect induced by the cryogenic coolant is dispersed throughout the body, thus, mitigating the work hardening effect. On the other hand, the continuous supply of the coolant during cutting of the alloy dispenses positive effects to the drill, whereas no coolant is provided during the cutting process in the between-the-holes mode of cooling depriving the tool of the associated benefits. The matter is different with the drilling of CFRP. The pre-process cooling of the drill is enough for a considerably weaker (in shear) work material to be drilled in exceedingly shorter processing time. Moreover, the impact of the cryogenic coolant on the work, in the continuous cooling mode, hardens it significantly due to its poor thermal conductivity. Therefore, the between-the-holes mode of cryogenic cooling is better than the continuous mode for the drilling of CFRP but not for Ti-6Al-4V.

The data on thrust force and specific cutting energy show similar patterns as both are dependent on work material’s shear strength. The latter is dependent also on cutting speed. Regarding the two performance measures, the between-the-holes cooling technique yields better results than continuous cooling for both the work materials. The underlying reason is that the cryogenic cooling of the work material during the cutting process enhances the strength of the work material causing it to draw more energy (and force) to get plastically deformed. The between-the-holes mode of cooling avoids such an anomaly because the work material is never impacted by the cryogenic fluid.

The between-the-holes cooling technique outperforms continuous cooling in respect of surface finish of the holes drilled in Ti-6Al-4V and the holes drilled in CFRP at the high level of cutting speed. The complex work expansion-contraction pattern induced by the application of a cryogenic fluid on the work material, as is the case in continuous cryogenic cooling, compromises the surface finish of the holes. Between-the-holes cooling is also found to yield better results regarding hole roundness, burr generation, and fiber pullout in CFRP drilling.

Finally, the continuous mode of cooling is better than between-the holes cooling regarding production time. The difference becomes more prominent at high levels of cutting speed, as is the case with hole-making of CFRP. On the other hand, between-the holes cooling is better than continuous cooling regarding consumption of the cryogenic fluid except when the cutting speed is very high. Reductions in production times and consumption volumes of a cryogenic fluid add to the economic sustainability of the machining process.

In context of the discussion provided above, it can be safely concluded that the between-the-holes mode of cooling is a far more effective way of applying a cryogenic coolant than the traditional mode of continuously supplying the fluid in drilling of CFRP. It outperforms the conventional approach with respect to almost all the performance measures of a machining process such as tool damage, cutting forces, cutting energy consumption, work surface quality, and consumption of the cryogenic fluid. The only area of concern for the novel cooling technique is increased production time. It is expected that the weakness in this regard can be minimized by increasing the flow rate of the coolant while reducing the time of drill’s exposure to the cryogenic fluid’s jet before cutting each hole. Regarding drilling of the titanium alloy, the choice of the cryogenic coolant’s delivery is not as straightforward. Although the between-the-holes cooling technique yields better results than the traditional approach in respect of cutting forces, specific cutting energy, holes’ surface roughness, and cryogenic fluid’s consumption cost, it is humbled against the continuous mode of cooling in respect of controlling tool damage. Longer production time is, obviously, another matter of concern but its percentage difference is much lower than in CFRP drilling. In this situation, the selection of the coolant’s supply mode hinges on the proportion of tooling cost in the total processing cost. A small proportion would tilt the decision in the favor of the between-the-holes mode of coolant’s delivery.

## 5. Conclusions

The presented work establishes the efficacy of a novel between-the-holes technique of applying a cryogenic fluid to the tool, without impacting the work, in hole-making of two commonly used materials: Ti-6Al-4V and CFRP. Its effectiveness is compared with the traditional approach of continuously supplying the coolant during cutting and dry drilling. The following conclusions can be drawn from the experimental results, their analyses, and discussion provided beforehand:

1. Between-the-holes cryogenic cooling effectively checks the progress of tool wear, more effectively than continuous cooling, in CFRP drilling. On the contrary, it underperforms in this regard in Ti-6Al-4V drilling owing to the higher shear strength of the titanium alloy and a much slower and coolant-less cutting process.
2. Application of the novel cryogenic technique yields lowers thrust force and specific cutting energy consumption in comparison to the continuous mode of cooling due to avoidance of work strengthening caused by the cryogenic fluid’s impact on the work material.
3. The between-the-holes cooling technique also leads to improvements in holes’ surface roughness, especially in the titanium alloy because of preventing complex work expansion-contraction patterns. It also yields significantly better surface quality at both sides of the holes in CFRP drilling.
4. The novel cooling technique though compromises productivity because of the additional time required to cool the drill, it generally consumes lesser cryogenic fluid than the continuous mode of cooling. Consumption of the cryogenic fluid is the only measure in which dry drilling outperforms the two cryogenic cooling options.
5. In a holistic view, the between-the-holes mode of coolant delivery is an unambiguous choice of heat dissipation for CFRP drilling. For Ti-6Al-4V drilling, the choice between the continuous and the between-the-holes modes of cryogenic cooling depends on the share of tooling cost in the total processing cost. A small share would lead to selection of the between-the-holes mode.

## Figures and Tables

**Table 1:** Mechanical properties of the CFRP and Ti-6Al-4V plates used in the study.

Material	Tensile Strength (GPa)	Tensile Modulus (GPa)	Elongation (%)	Compressive Strength (GPa)	Yield Strength (GPa)	Shear Strength (GPa)	90° Tensile Strength (GPa)
CFRP	2.35	140	1.9	1.57	2.24	0.09–0.16	0.086
Ti-6Al-4V	1.004	115	15	0.903	0.928	0.57	–

**Table 2:** Production time and LN_2_ consumption for cutting 5 holes in each of the 12 runs.

S/No.	Work Material	*V*_c_ (m/min)	Cooling Option	Production Time (sec)	LN_2_ Consumption (L)
1	CFRP	100	Dry	19.7	0
2	CFRP	100	Continuous	19.7	0.20
3	CFRP	100	Between-the-holes	44.7	0.17
4	CFRP	150	Dry	14.0	0
5	CFRP	150	Continuous	14.0	0.15
6	CFRP	150	Between-the-holes	39.0	0.17
7	Ti-6Al-4V	13	Dry	113.0	0
8	Ti-6Al-4V	13	Continuous	113.0	0.98
9	Ti-6Al-4V	13	Between-the-holes	158.0	0.33
10	Ti-6Al-4V	20	Dry	74.3	0
11	Ti-6Al-4V	20	Continuous	74.3	0.66
12	Ti-6Al-4V	20	Between-the-holes	119.3	0.33

## Data Availability

Data sharing not applicable.
